# Mechanical Behaviour and Impact of Various Fibres Embedded with Eggshell Powder Epoxy Resin Biocomposite

**DOI:** 10.3390/ma15249044

**Published:** 2022-12-17

**Authors:** Aburpa Avanachari Sivakumar, Sankarasabapathi Sankarapandian, Siva Avudaiappan, Erick I. Saavedra Flores

**Affiliations:** 1 Department of Mechanical Engineering, Varuvan Vadivelan Institute of Technology, Dharmapuri 636701, India; 2 Department of Mechanical Engineering, Alagappa Chettiar Government College of Engineering and Technology, Karaikudi 630003, India; 3 Departamento de Ingeniería Civil, Universidad de Concepción, Concepción 4070386, Chile; 4 Centro Nacional de Excelencia para la Industria de la Madera (CENAMAD), Pontificia Universidad Católica de Chile, Santiago 9170201, Chile; 5 Departamento de Ingeniería en Obras Civiles, Universidad de Santiago de Chile, Santiago 9170201, Chile

**Keywords:** fibres, eggshell powder, sustainability, strength, epoxy resin

## Abstract

Natural fiber composites are becoming an alternate material to synthetic fiber composites, and the use of eggshell bio-filler has been explored in polymer composites as environmental protection. Jute, coir, and sisal fibers were utilized in this research to make composites out of natural fibers. Polymer composites were made using epoxy resin with different amounts of eggshell powder (ESP) as fillers (2%, 4%, 6%, 8%, and 10% of weight). The mechanical and biodegradability properties of the synthesized composites were investigated. The testing results showed that composites with an optimum percentage of 6% ESP as filler improved mechanical characteristics significantly in all three fiber composites. Among the three fibers, coir fiber with 6% ESP added showed a substantial increase in tensile, flexural, impact, and hardness strength properties by 34.64%, 48.50%, 33.33%, and 35.03%, respectively. In addition, the percentage weight loss of coir fiber composites at 9 weeks is noteworthy in terms of biodegradability testing. As a result, epoxy composites containing eggshell fillers could be employed in applications requiring better tensile, flexural, impact, and hardness strength.

## 1. Introduction

The manufacturing sector is shifting toward more environmentally responsible and sustainable methods of production as a result of the rapid advancements in science and technology. There is a plethora of natural fibres to choose from, and they can be sourced from just about everywhere. Natural fiber-based composites are rapidly replacing synthetic fiber-oriented composites as they are preferred for their superior biodegradability, renewability, decomposability, rigidity, higher length to weight ratio, and low cost. A composite is a material made from a combination of two or more materials that exhibits improved characteristics and properties over either of the constituent materials when used alone [1]. Hybrid composites of natural and synthetic fibres have attracted increased attention from industries in recent years as a means of reducing weight and being more environmentally friendly [2]. Overexploitation and overconsumption of non-renewable resources have negative effects on the environment, and the use of fossil-based composites is on the rise around the world. As a result, energy scarcities may worsen as demand for power is expected to double in the next two decades [3]. Therefore, it is crucial to manage the energy demand that limits the damage done to the environment. Greenhouse gas emissions can be reduced by cutting back on energy use, which is where energy efficiency comes in [4]. Calcium carbonate, which is water repellent, is the most often utilized as an inorganic filler in composite production today. CaCO_3_ is most easily obtained from chicken eggshell, which is abundantly produced as a waste material each year. In the United States, the Environmental Protection Agency (EPA) ranks eggshell waste 15th among wastes created each year, and European Commission laws classify eggshell as the most hazardous waste [5]. Poultry, food production companies, residences, bakeries, and restaurants are the primary sources of eggshell waste [6]. As a result of waste management being a difficult challenge, scientists are placing a premium on garbage disposal. Because eggshells are economical and light in weight, they are used in load-bearing materials such as the automotive sector and other structural applications [7]. As a result, several researchers have investigated the use of this waste chicken eggshell as a reinforcement in a range of composites in order to improve the strength of the composites and, as a result, discover a means for its disposal [8].

Polymeric composites may help to improve energy efficiency because of their inherent properties. When used in automobiles, composites can help in reduction on weight and fuel costs [9]. Therefore, it is expected that the use of biocomposites in the automobile industry will be facilitated by the demand for fuel consumption, weight reduction, and emission control. In composites, reinforcement is typically made from naturally occurring fibres because they are abundant in nature and can be processed in a more eco-friendly manner [10]. Natural fibres are widely available, biodegradable, and harmless to human and animal health. In addition, natural fiber-reinforced fibres are regarded as a viable alternative to petroleumand fossil-based fibres in the future. In emerging nations like Vietnam, Thailand, and India, natural fibres like jute, coir, banana, and sisal are abundant [11]. In recent decades, engineering applications involving polymer composites reinforced with natural fibres have increased significantly due to the composite’s advantageous properties, as well as the fiber’s and the material’s environmental friendliness [12]. Starch, a form of natural polymer, has been viewed as a potentially useful input in the production of biocomposites. Previous researchers have analysed the effects of digestate (DG) sludge from agricultural biogas plants on the physicochemical and mechanical properties of thermoplastic starch materials [13].

In recent years, an increasing number of scholars have focused on environmental pollution and limited petroleum resources. The use of natural fibres in scientific research is increasing daily. Natural fibres like bamboo, sisal, kenaf, coir, and jute have properties that make them ideal for use in composite components due to their lightweight and inexpensive nature [14]. These fibres have high calorific value, can be burned to recover energy after their useful life is over, and are renewable. As plants contain so much cellulose, their fibres absorb water easily, limiting their usefulness as a construction material [15]. At a lower density, natural fibres could achieve superior characteristics such as strength, stiffness, and tenacity [16]. Consequently, natural fibres could serve as substitutes for synthetic fibres. Some studies have looked into hybridizing banana fiber-reinforced composites with other nano additives like nanoclay [17] and nanosilicon [18] to boost their mechanical properties. When banana fibre was mixed with nanoadditives, the hydrogen bonds of nanoclay formed at the fiber–matrix interfaces, which boosted the material’s mechanical strength [18].

Jute, among the commercially available crop fibres, has an exceptionally high proportion of stiff natural cellulose. The content of cellulose can be reduced by surface leaching for a specified time through chemical and physical treatments of the fibre and filler [19]. This has led to its use in composites made with epoxy and polyester resins [20]. Banana-hemp-glass-reinforced hybrid composites, hemp, and glass fiber-reinforced banana fibres were created and tested by Bhoopathi et al. for their tensile strength, flexural strength, and impact strength, among other mechanical properties. According to the results, this epoxy composite with hemp-glass fibre reinforcement can be used in place of synthetic fibre composites [21]. Cellulosic fibres make up coir, and hemi-cellulose and lignin serve as the fibrebinding agents. Because coir fibre has a relatively high lignin concentration compared to other natural fibres, it becomes stiffer, tougher, and more durable.

A coir fiber-reinforced polypropylene composite panel for car interior applications was explored by Ayrilmis et al. According to this study, coir fibre has the ability to reinforce thermoplastic composites, particularly when used to partially replace expensive and heavy glass fibres [22]. Cementitious composites using coir fibre boosted their flexural toughness and flexural toughness index by a factor of more than 10% [23]. Many scientific researchers have focused at the mechanical properties of composite materials including how different nanofillers affect them [24,25,26]. Due to its great qualities, sisal is a strong, lightweight, natural polymer, and strong material utilized in a variety of sectors as a composite material. The mechanical properties of epoxy composites made by hand layup with untreated and treated Portunus sanguinolentus shell powder (10 wt.%) and jute were evaluated. Sodium hydroxide-treated Portunus sanguinolentus shell powder-filled jute fabrics demonstrate enhanced mechanical capabilities as a result of filler and fibre matrix bonding [18].

The extensive literature research makes it abundantly evident that both organic and inorganic fillers have an ideal weight proportion of fillers in the fiber-reinforced composites to improve their mechanical properties. It has been found that no research has been conducted on the use of eggshell powder as a filler in epoxy composites that also include other fibres including jute, coir, and sisal. The current work proposes developing and studying hand layup compression epoxy composites reinforced with jute, coir, and sisal with and without eggshell powder at varied weight percentages of filler material. Our primary objective has been to create successive commercially viable sustainable products using natural fibres. In this research paper, we used jute, coir, and sisal fibre as reinforcement and eggshell powder as filler with epoxy resin to produce a hybrid composite material. We conducted the necessary experiments and analyses to demonstrate the composite’s viability with natural fibres.

## 2. Experimental Programme

### 2.1. Materials

#### 2.1.1. Eggshell Powder Preparation

**Figure 1:**
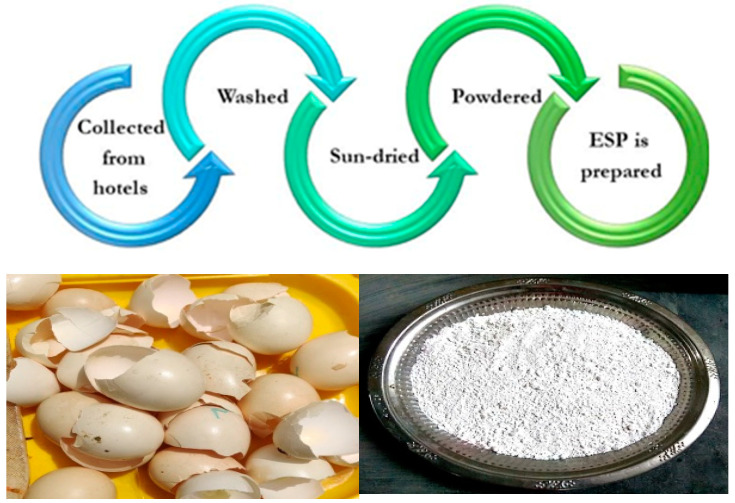
Method of eggshell powder manufacturing.

**Figure 2:**
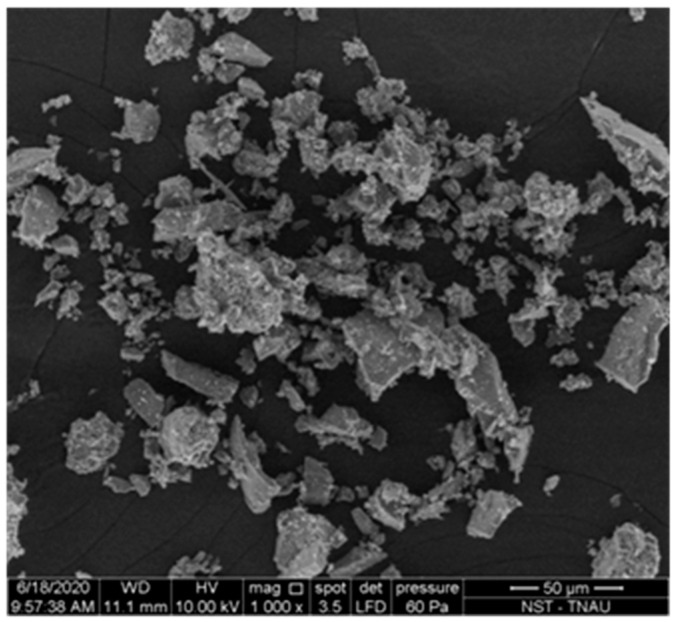
SEM Image of eggshell powder.

Ground-up eggshells were used as filler. Polymers can benefit from fillers in a number of ways, including improved strength, surface texture, hardness, wear properties, and cost. The shells of eggs are typically thrown away in kitchens, factories, homes, etc. Used in conjunction with resin, it serves as an effective filler material that also provides sturdy support for the reinforcement of fibres. Eggshells were collected from local hotels inNamakkal, Tamilnadu, India. The samples were washed with water, making sure to removethe white membrane inside the eggshells and then sun-dried. The eggshells were then grounded into powder using kitchen mixer and sieved with a 90 µm mesh sieve. Figure 1 represents the method of eggshell powder manufacturing. Figure 2 shows an SEM image of eggshell powder, which reveals that the powder consists of tiny, irregular crystals after being finely ground.

#### 2.1.2. Fibres

**Figure 3:**
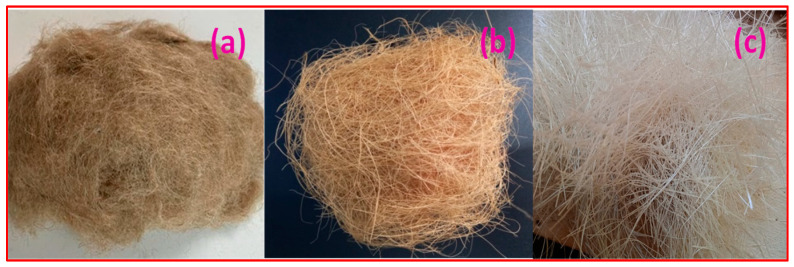
Fibres utilized: (**a**) Jute; (**b**) Coir; (**c**) Sisal.

The most popular natural fibres are made from the exterior stems of specific plants, including sisal, jute, flax, hemp, and coconut. Natural fibres are becoming more popular due to their extremely low weight, acceptable structural performance, and “green” characteristics like recyclable nature. With regard to the latter, they are more affordable (less energy is used in their manufacture) and sustainable (biodegradable and renewable). Additionally, they have the lowest density of any structural fibre, although they are strong and rigid enough for specific applications. The fibres used to produce the sandwich composites in the current study were of Jute, coconut and sisal fibre. The fibre used in the current study is procured from the supplier Go Green products, Valasarvakkam, Chennai, Tamilnadu, India. The fibre is shown in Figure 3 and the properties of each fibre are listed in Table 1.

#### 2.1.3. Epoxy Resin

Epoxy resin is a good adhesive for the materials utilized in this study. It is also considered a polyepoxide, a reactive class of pre-polymers and polymers that contains epoxy groups. Epoxides are used in fiber reinforcement as they produce stronger and temperature-resistant composite parts. Whilst some epoxy resin/hardener combinations will cure at ambient temperature, many require heat, with temperatures up to 150 °C being common, and up to 200 °C for some specialist systems. Insufficient heat during cure will result in a network with incomplete polymerization, and thus reduced mechanical, chemical, and heat resistance. The cure temperature should typically attain the glass transition temperature (Tg) of the fully cured network in order to achieve maximum properties. The temperature is sometimes increased in a step-wise fashion to control the rate of curing and prevent excessive heat build-up from the exothermic reaction. The epoxy resin (LY556) and hardener (HY 951) is procured from Herenba Instruments&Engineers suppliers, Chennai, India. Theproperties of epoxy resin LY 556 are listed in Table 2.

### 2.2. Processing

#### 2.2.1. Matrix Preparation

The matrix was prepared by varying the preferred percentage of the eggshell powder as a filler material. Six different compositions of the matrix (0% ESP, 2% ESP, 4% ESP, 6% ESP, and 10% ESP)with eggshell powder were prepared from 0% to 10%. The filler material was increased by 2%for each composition in the resin.

#### 2.2.2. Specimen Preparation

**Figure 4:**
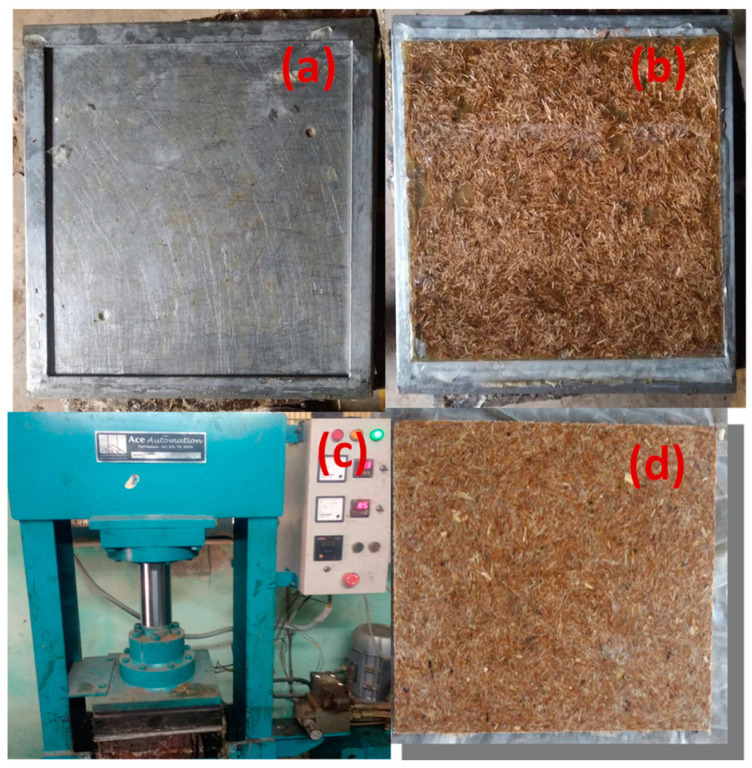
Composite fabrication process: (**a**) Fabrication mould; (**b**) Laying process; (**c**) Pressing process; (**d**) Fabricated composite specimen.

The specimen was prepared from the composite reinforced with jute, coconut, and sisal fiber, which was fabricated to a size of 300 mm × 300 mm. The composite was prepared using the hand lay-up method. The samples were prepared in a mold made of mild steel with dimensions of 300 mm (length) × 300 mm (width) and 4 mm thickness. A mixture of 10:1 was prepared with epoxy and hardener, respectively, based on the volume percentage required to customize a matrix. The filler material wasadded in epoxy resin and mixed with the help of a glassrod uniformly. After thorough mixing of epoxy and filler, hardener is added and mixed well till a uniform mixture is obtained. The resin was coated over the sheet spread on the mold. The fibers are placed over the epoxy coating. The distribution of resin was even on the mold covering the total area of the fiber. The fiber material was stacked and the remaining epoxy resin matrix along with hardener mixture was poured on to the mold. The composite material is allowed to cool down completely and later pressed down to remove air bubbles, if any. It was then left to cure at room temperature and was finally removed from the mold for testing its mechanical and thermal properties. Specimens are then cut by using a handsaw or jigsaw as per the ASTM standards for the various tests. Three identical test specimens were prepared for different tests. The process of making a composite is shown in Figure 4.

## 3. Tests conducted

### 3.1. Mechanical Properties Test

Various tests on mechanical properties such as the tensile test, flexural test, impact test, and hardness testwere conducted. The tensile and flexural strength of the specimen was done in TINIUS OLSEN H10KT universal testing machine (Tinius Olsen, Ltd., Noida, India) having a frame capacity of 10 kN. Using a universal testing machine that meets ASTM D 638, the tensile behaviour of samples is tested. Samples that are 250 mm long, 25 mm wide and 2.5 mm thick were used for testing. The sample was put between the two grips of a universal testing machine (UTM) with an electronic extensometer that could be adjusted by hand and had a 60 KN capacity. The tensile strength of the composite was found by taking the average of the three times each test was done. The flexural strength is measured using a three-point bend test. Test specimens measuring 130 mm × 25 mm × 3.2 mm were utilized. This procedure measures the flexural characteristics of fiber-reinforced polymer composites. The flexural strength is computed in accordance with ASTM D 790. The composites’Charpy impact strength was evaluated with an industry-standard impact machine in accordance with ASTM D256. The test used the typical 55 mm long, 10 mm x 10 mm cross-sectional test specimen with a 450 V-notch and a 2 mm depth. The impact strength was determined by doing each test three times and averaging the results. The Brinell hardness of the specimen was measured with a conventional Brinell hardness tester as per ASTM D 2240. A 5 mm hard metal ball indenter was used to apply a force of 250 kg for 30 s to the specimen, and the indentation diameter was measured using a microscope. The hardness of the specimen was measured in three locations, and an average was calculated.

### 3.2. Biodegradable Test

Biodegradation is the process by which a molecule decomposes due to enzymes or compounds released by bacteria or fungi in soil [27]. The soil burial method (ASTM D5988/D5338) is used to assess the biodegradability of materials, and the results are represented as weight loss (%) [28]. The rate of biodegradation caused by moisture and microorganisms throughout the soil burial period can be calculated from the weight loss of the buried material [29]. Analyzing the biodegradability of the composite specimens, the soil burial test was undertaken. In order to evaluate the biodegradation of the samples, the initial weight (W1) of the samples was measured before they were buried in the soil. After 2, 4, and 9 weeks, samples were extracted from the soil and the weight of the samples wasrecorded (W2). Using Equation (1), the weight loss of the sampleswasdetermined. Sample deterioration was quantified by measuring how much weight had been lost.

Weight Loss (%) = ((W1 − W2)/W1) × 100(1)

where

W1 = initial weight of the samples before soil burial test, g
W2 = weight of the sample after soil burial test, g

## 4. Results and Discussion

### 4.1. Tensile Strength

**Figure 5:**
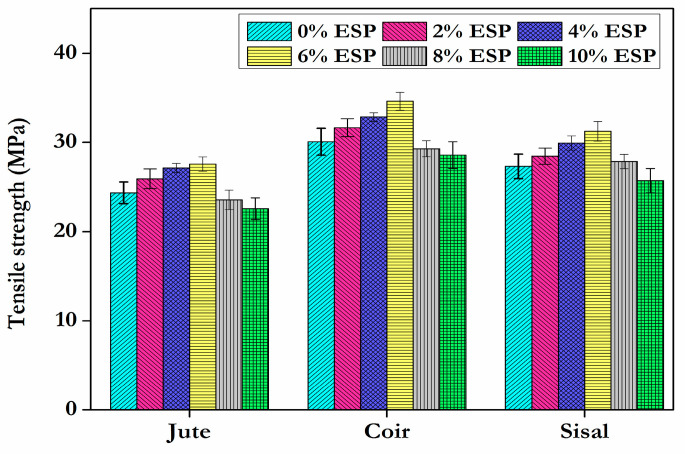
Tensile strengthof composite specimens with different fibres.

**Figure 6:**
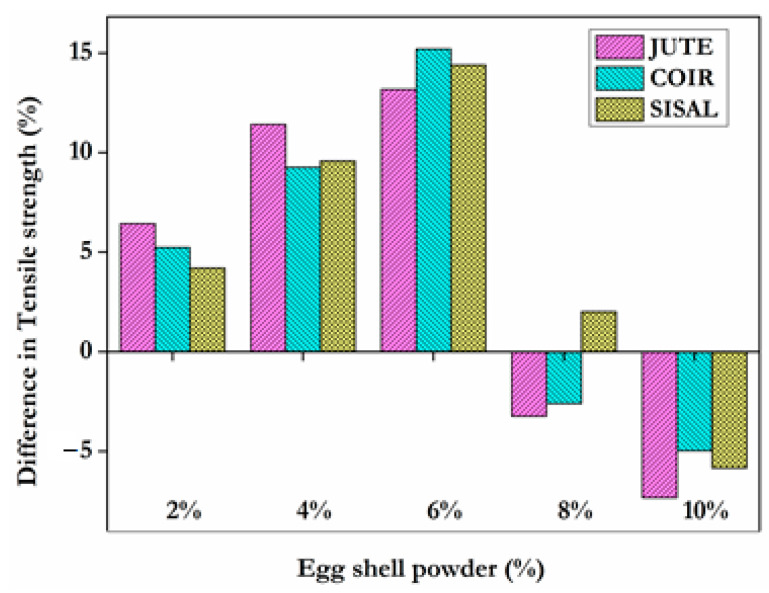
Percentage difference of tensile strength with ESP.

The tensile test enables the selection of an acceptable material for a certain application, as well as the control of the specimen’s quality and behaviour under various types of loads. Without any hybridization, the epoxy/jute fibre composite, epoxy/coir composite, and epoxy/sisal composite recorded tensile values of 24.36 MPa, 30.07 MPa, and 27.31 MPa, respectively. The epoxy/jute composite had the lowest recorded tensile strength. Figure 5 depicts the tensile strength of different fibre composites. The testedresults revealed that composites with 2%, 4%, 6%, 8%, and 10% of filler material with jute carried loads of 25.93 MPa, 27.14 MPa, 27.57 MPa, 23.57 MPa, and 22.57 MPa, respectively. Composites with the same proportions of filler material with coir fibre carried loads of 31.64 MPa, 32.86 MPa, 34.64 MPa, 29.29 MPa, and 28.57 MPa. Composites with the same proportions of filler material with sisal fibre carried loads of 28.46 MPa, 29.93 MPa, 31.24 MPa, 27.86 MPa, and 25.71 MPa. As the eggshell powder and epoxy resin were mixed together, the tensile strength increased gradually. It is obvious from Figure 6 that the strength begins to increase and then decreases as the amount of eggshell powder used as filler material increases. Comparatively to the other three forms of fibre, coir displayed a high tensile load of 34.64 MPa at the optimal dose of 6% eggshell powder as filler. Figure 6 depicts the percentage difference of tensile strength for the composites with eggshell powder.

**Figure 7:**
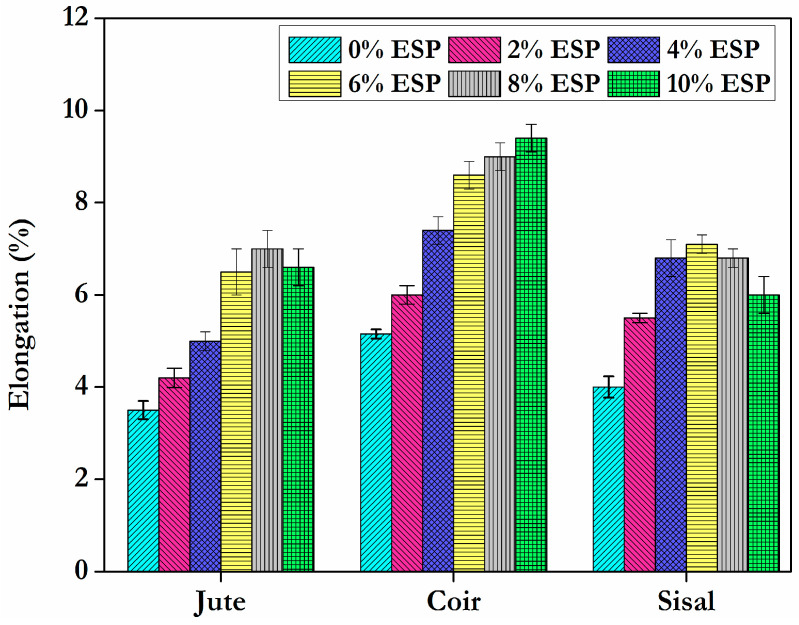
Elongationat tensile strength for tensile specimens.

From the experimental examination, the optimal filler material concentration has been determined. When the amount of eggshell powder in a composite matrix exceeds 6%, the strength begins to decrease. This may be the result of eggshell powder aggregating in the fibres. Agglomeration indicates inadequate filler dispersion in the matrix. These agglomerates are sites of stress concentration that facilitate the initiation and propagation of cracks, resulting in brittle failure [30]. It can be attributed to the decrease in the binding strength of the resin at increasing eggshell powder loading and a similar observation was explored by Ashokumar et al. [31]. At a dose of 6% eggshell powder, the percentage of the tensile strength of the fibres jute, coir, and sisal increases by 13.2%, 15.2%, and 14.38%, respectively, compared to composites with no eggshell powder. In addition, the fibres can be folded and there is no link between the folded and unfolded portions, resulting in a decrease in strength. Entanglement of fibres may also contribute to a decrease in strength. The percentage elongation of composites with various fibres is shown in Figure 7.

### 4.2. Flexural Test

**Figure 8:**
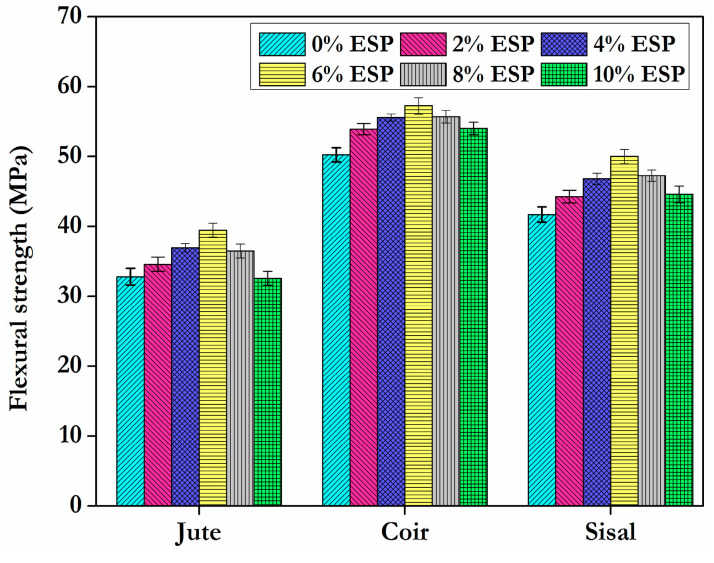
Flexuralstrength of composites.

The breaking load of the flexural strength is represented in Figure 8. Figure 8 shows that a composite containing 6% ESP as filler bears the highest load in all three types of fibres. The tested results revealed that composites with 2%, 4%, 6%, 8%, and 10% of filler material with jute carried loads of 36.30 MPa, 38 MPa, 38.60 MPa, 33 MPa, and 31.60 MPa, respectively. Composites with the same proportions of filler material with coir fibre carried loads of 44.30 MPa, 46 MPa, 48.50 MPa, 41 MPa, and 40 MPa. Composites with the same proportions of filler material with sisal fibre carried loads of 39.84 MPa, 41.90 MPa, 43.74 MPa, 39 MPa, and 36.12 MPa. Comparing the composites without ESP, composites with an addition of ESP improved the flexural strength. The flexural strength of composites increased gradually with the addition of ESP and then it decreased.

The higher ultimate strength indicates that the coir fibre has superior interfacial bonding with epoxy and reveals the ability of eggshell powder to improve the flexural behaviour of the hybrid composite. The increase in flexural strength is due to the surface interaction of the fibre with the matrix and filler material, which improves wettability inside the composite and hence minimizes fibre pullout when testing. Although there is not a substantial variation in the ultimate strength of sisal fibre composite specimens, significant differences have been seen in the composite specimen with jute fibres. Furtheraddition of eggshell powder decreases the flexural strength. It could be attributable to the resin’s diminished covalent connection when the eggshell powder concentration rises and similar results was observed [31]. When compared to glass fiber, which currently accounts for the majority of automotive composites, natural fibers have the potential to reduce vehicle weight by up to 40% net vehicle weight [32].

### 4.3. Impact Test

**Figure 9:**
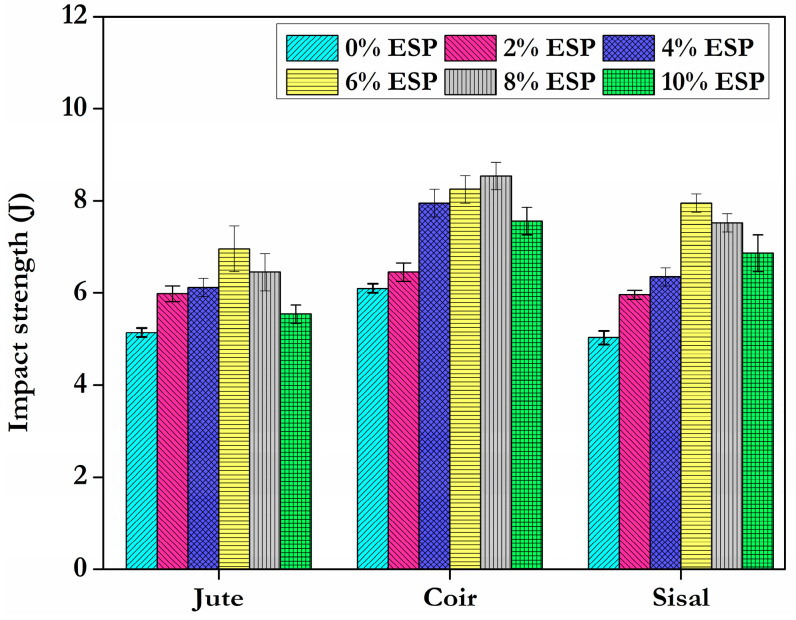
Impact load value of different fibres with varying proportions of ESP.

The impact strengths of the specimens using composites of different fibre types and varying amounts of eggshell powder are shown in Figure 9. For comparison, coir fibre has an impact strength of 8.5 J when combined with 8% ESP, sisal fibre has impact strength of 8.1 J when combined with 6% ESP, and jute fibre has an impact strength of 7.2 J when combined with 6% ESP.The impact strength is determined by the amount of deformation that occurs prior to failure, in addition to the interfacial interaction between the filler and polymer. Since materials with high strength and modulus fail at low deformations, the lower impact strength of jute fibre is to be expected.

During an impact test, the particles in the polymer matrix are thought to increase the likelihood of microcrack formation and propagation throughout the matrix, thereby disrupting the polymer-filler interface [33]. Impact strengths for the hybrid composites in this research are higher than those previously reported for synthetic non-biodegradable epoxy resin and natural biodegradable epoxy resin. Improved fiber–matrix interaction is responsible for the greater impact strength. Based on these findings, it appears that coir fibre addition is a viable option for enhancing impact strength. This is due to the fact that coir fibres are naturally flexible, and the addition of a bioepoxy matrix and filler material system increases their ability to soak up impact energy.

### 4.4. Hardness Test

**Figure 10:**
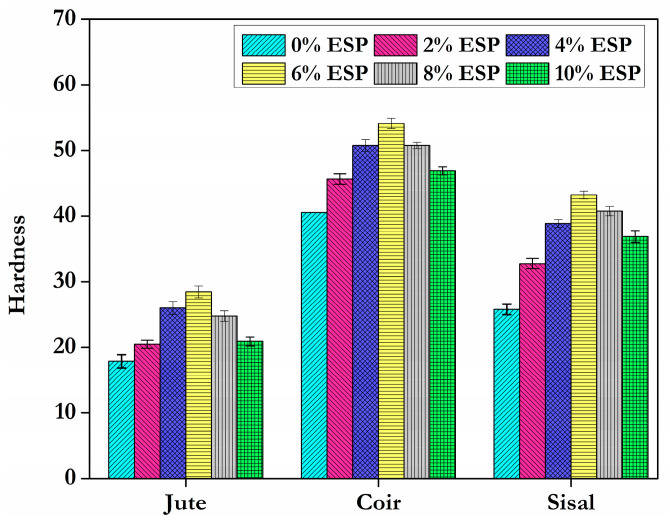
Hardness of different fibres with varying proportion of ESP.

The behaviour of hardness in relation to the addition of eggshell powder filler parallels the trend observed in tensile strength tests. ESP increases the hardness of various fibre epoxy composites. Compared to pure epoxy without filler, the addition of 2 wt.% ESP filler in jute, coir, or sisal fibre increases the hardness by 14.44%, 12.48%, and 27.08%, respectively. Further addition (4%, 6%, 8%, 10%) of all fibres increases the value of hardness. The hardness values of all composite specimens are shown in Figure 10. Effective dispersion of the ESP filler in the epoxy matrix of the reinforced composite matrix is responsible for the increased hardness of the investigated ESP filler reinforced epoxy composites. Compared to sisal and jute fibre composites, the highest hardness value is achieved by coir fibre epoxy resin composite with a 6% weight addition (Figure 10). All fibre composites exhibited a slight increase in hardness with additions above 2%. However, there is a decrease in hardness value above 6% ESP filler content in the composite.

### 4.5. Soil Burial Test

**Figure 11:**
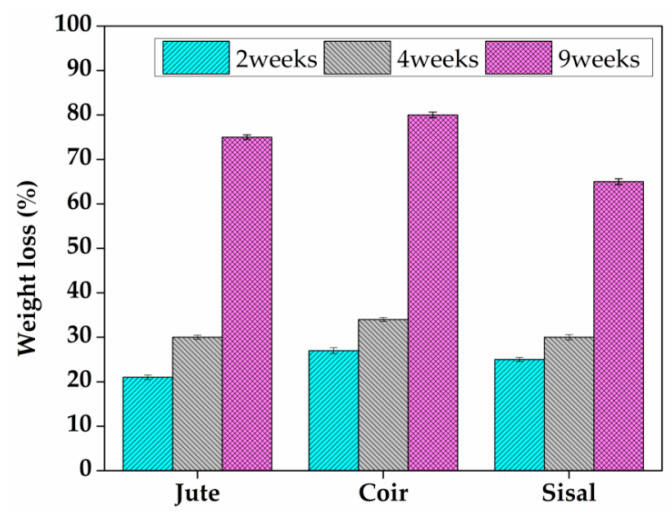
Weight loss after soil burial at 6% of eggshell powder.

By monitoring the amount of material lost in weight over time, it is possible to assess the rate of biodegradation brought on by soil moisture and microorganisms [34]. Figure 11 depicts the weight loss over 2, 4, and 9 weeks of burial at 6% of eggshell powder. Compared to two and four weeks, all composites lost more weight after nine weeks of burial. This is due to the fact that the material lost more weight because more microbes were active throughout the longer time the material was buried [35]. Comparing all the fibres the degradation percentage for coir fibre is high when compared jute and sisal fibre at all times. This occurrence may be attributable to the greater hydrophilicity of fibre. At 9 weeks of soil burial with respect to coir fibre, the highest weight loss of about 80% is obtained.

## 5. Conclusions

Globally, there is a greater emphasis placed on materials that are renewable, biodegradable, compostable, and sustainable. In this research, composites are made by reinforcing epoxy LY556 polymers with natural fibres like jute, coir, and sisal and then filling them with eggshell powder.

- For all the fibre composites, the best amount of eggshell powder was found to be 6% by weight. The composites’ tensile strength and modulus increase as the filler loading increases from 2% to 10% wt.
- Increasing the filler quantity reduces the strength in all three types of fibres as the fiber and matrix bonding area decreases. The results show that 6% eggshell powder has good tensile properties and impact properties.
- The biodegradability of the fibres is improved with addition of time duration. This work aims to reduce the amount of eggshell waste that ends up in landfills. It will also try to find a replacement for eggshells in advanced engineering applications like automotive, aerospace, etc.
- Better mechanical properties are anticipated with homogeneous dispersion of filler particles in the matrix and good compatibility and adhesion of the fibre, matrix, and filler. Tribological studies using eggshell as fillers are still being conducted. As a result of this study, it is clear that there is a potential for using eggshell particle as a filler in composites and studying their wear behavior so that it can be employed in future structural and automobileapplications.

## Figures and Tables

**Table 1:** Properties of fibres.

Type of Fibre	Tensile Strength (MPa)	Youngs Modulus (GPa)	Elongation at Break (%)	Density (g/cm^3^)
Jute	510–710	26.5	1.6	1.21
Coir	825–880	8 –15	15	1.40
Sisal	610–820	9–22	2–3	1.34

**Table 2:** PropertiesofEpoxy resin LY556.

S.No	Properties	Values
1	Transition strength (°C)	120–130
2	Tensile strength (N/mm^2^)	85
3	Tensile modulus (N/mm^2^)	10,500
4	Elongation at break (%)	0.8
5	Flexural strength (N/mm^2^)	112
6	Flexural modulus (N/mm^2^)	10,000
7	Compressive strength (N/mm^2^)	190

## Data Availability

Not applicable.
